# Wind farms dry surface soil in temporal and spatial variation

**DOI:** 10.1016/j.mex.2023.102000

**Published:** 2023-01-02

**Authors:** Gang Wang, Guoqing Li, Zhe Liu

**Affiliations:** a School of Resources and Environmental Engineering, Ludong University, Yantai, Shandong Province, 264025, China; b Ecological Monitoring Department, China National Environmental Monitoring Centre, Beijing, 100012, China

**Keywords:** Wind turbine, Soil moisture, Drought, Seasonal, Study method of quantitative influence of wind farm on soil moisture

## Abstract

Wind farms have been proved to have potential impact on the ecology. As an important ecological factor, soil moisture has a great impact on the ecosystem. Therefore, it is of great significance to explore the effect of wind farms on soil moisture. At present, the remote sensing data can be used to calculate the soil moisture of wind farm conveniently, but its spatial resolution is poor. Moreover, the measured soil moisture can't express the spatial difference. Therefore, through the effective combination of remote sensing data and measured data, this method can accurately judge the impact of wind farm on soil moisture. This method investigated wind farms located in the grasslands of China. Remote sensing images and field data were used to explore the area and extent of influence of wind farms on grassland soil moisture. We use Landsat images and field measurements to derive a linear relationship between the soil moisture and the TVDI, which was calculated based on the land surface temperature and NDVI, was developed in this work. The correlation was used to reverse spatial distribution map of soil moisture before and after the construction of wind farms. The diurnal and seasonal variation of the influence of the wind farm on the grassland soil moisture was also judged.

• This method of combining measurement and remote sensing provides a reference for analysing the influence of wind farms on soil moisture.
• This method can be used for reference to compare the meteorological factors of different wind directions before and after the construction of wind farms.

## Graphical abstract

**Figure:**
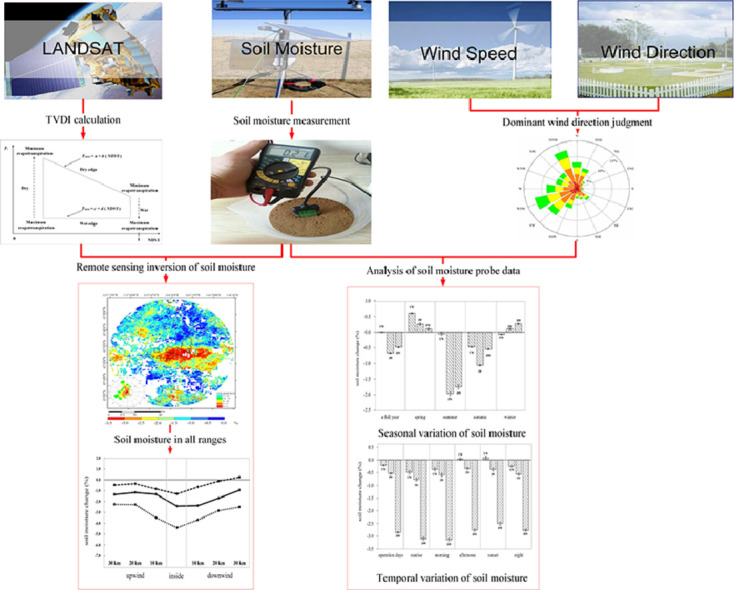

Specifications table

**Table:** 

Subject Area:	Environmental Science
More specific subject area:	Climatology
Method name:	Study method of quantitative influence of wind farm on soil moisture
Name and reference of original method:	The TVDI is a simplified land surface dryness index based on the relationship between the land surface temperature (Ts) and the normalized difference vegetation index (NDVI).
Resource availability:	https://data.mendeley.com/datasets/39cfrfr2w9/1

## Background

With the continuous growth of the global economy, demand for energy is increasing, with increasing demand for renewable sources to mitigate climate change [1]. Wind energy resources are distributed across the world, and the technology proven and cost-competitive. Although wind farms are widely considered ‘green energy’ they still impact the environment. Ecosystem impacts during construction and operation have been widely reported, including the potential to reduce, fragment, or degrade habitat for wildlife, fish, and plants. However, the impacts on local climate, and thus longer-term impacts on ecosystems are less well understood. Soil moisture changes are important for nutrient and energy cycling in ecosystems and have a direct impact on local wildlife. Xie (2015) used the measured data to monitor the effects of wind farms on vegetation and soil in low-water, semi-arid regions. After the wind farm is built, there is no consistent trend in soil moisture based on wind direction or distance from the wind farm (0–100 m) [2]. Armstrong et al. (2016) used observation data from a wind farm in Scotland to judge the impact of wind turbine operation on the local climate in podzolic soil. They found that there was no significant trend in changes in soil moisture (to a depth of 10 cm) caused by the operation of wind turbines, which may be related to the high water table at the wind farm [3]. Tang et al. (2017) used 0.25° × 0.25° climate change initiative (CCI) soil moisture data derived from MODIS data to judge the impact of wind farms on vegetation in Texas and Illinois, USA. By comparing the annual soil moisture difference between the wind farm and non-wind-farm regions, it was found that the wind farm soil moisture reduced by 0.3% after construction in the desert and yellow-brown soils [4]. With remote sensing data, although the soil moisture before and after a wind farm has been built can be calculated, the spatial resolution of the data is low and cannot be effectively combined with field-measured data. Therefore, many difficulties remain in accurately judging the impact of wind farms on soil moisture.

Therefore, this paper investigated wind farms located in the grasslands of China. Remote sensing images and field data were used to explore the area and extent of influence of wind farms on grassland soil moisture.

## Purpose

(1) To use Landsat images and field measurements to derive a spatial distribution map of soil moisture before and after the construction of wind farms.
(2) To compare spatial differences in soil moisture before and after construction of wind farms and judge the extent of influence of wind farms on soil moisture under different wind directions.
(3) To characterize seasonal and daily variations in soil moisture and evaluate the impact of seasonal and daily differences of wind farms on soil moisture.

## *Method details

### Measured and meteorological station data

**Fig. 1:**
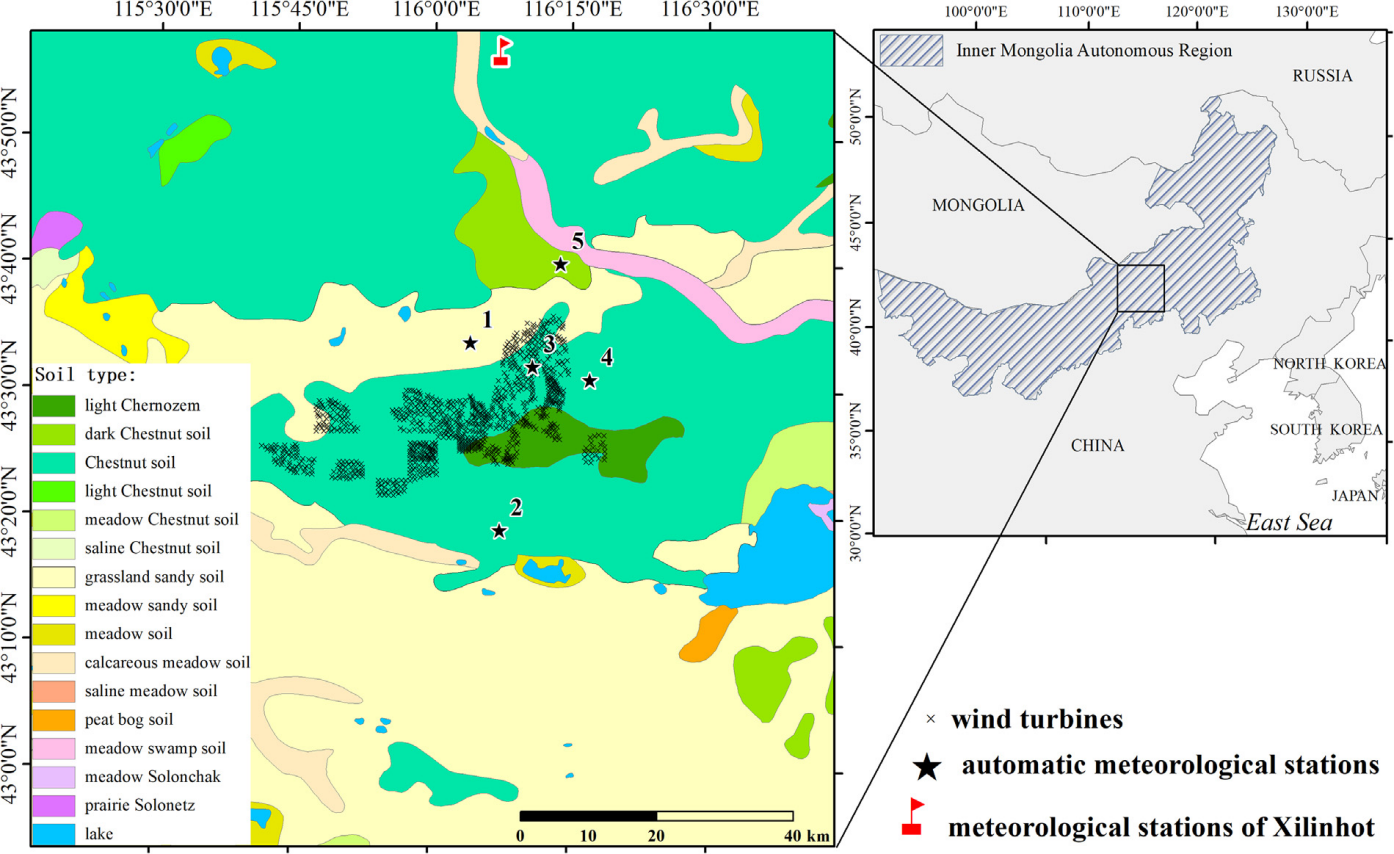
Location of the study area.

Automatic meteorological stations (China Wuhan Zhongke Nenghui Technology Development Co., Ltd.) were installed in five different locations (Fig. 1). The depths of soil temperature and moisture probes are 52 mm. The soil moisture measurement range was 0–100% (by volume) with an average error of 1% in the range 0–53% and 2% in the range of 53–100%. The temperature measurement range (± error) was −40–80 °C (± 0.4 °C). An NHFS45BP wind speed sensor (accuracy of ± 3% and resolution of 0.1 m/s) at 1.8 m, and an NHFX46AU wind direction sensor (accuracy of ± 3° and resolution of 1°) at 1.8 m were used.

### Experimental acquisition of the soil moisture

**Fig. 2:**
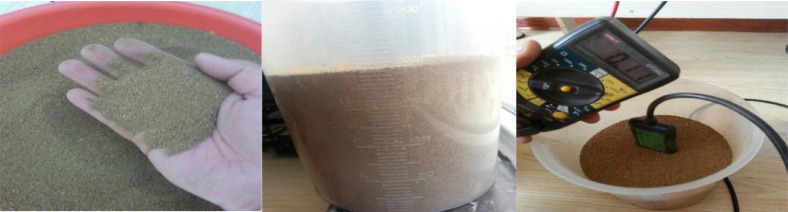
Preparation and results of the initial soil moisture experiment.

With a large number of cups, 2000 ml of dry soil was taken. After the measurement, a voltage output of 0.1 V was acquired, which corresponds to 5% moisture. Thus, it can be argued that in of the measured soil sample, there are 100 ml of water (Fig. 2).

**Fig. 3:**
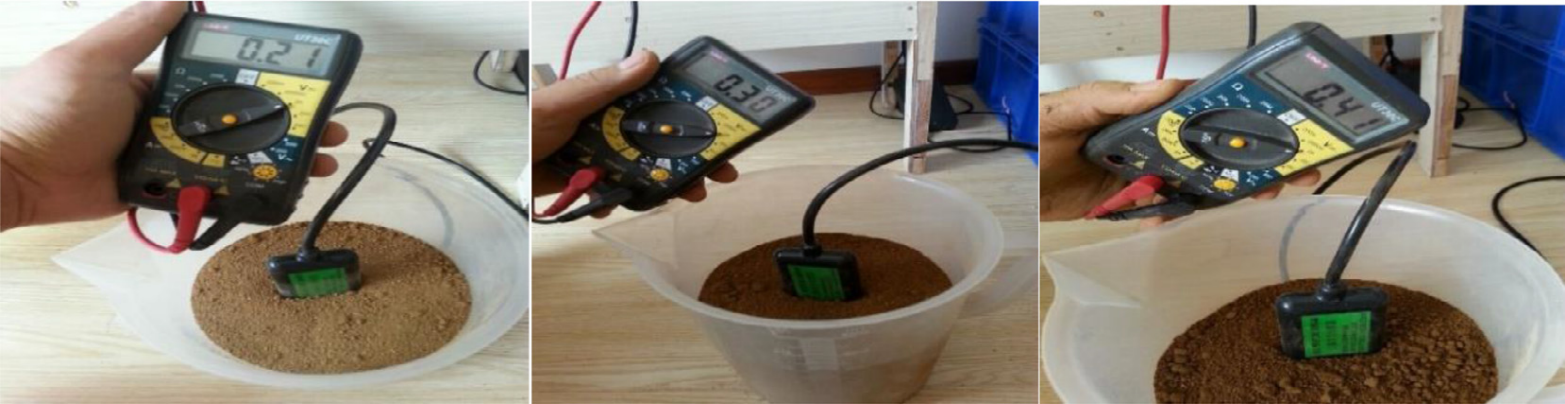
Soil moisture changes after adding 100 ml of water each time.

Next, 100 ml of water were added to the cup each time. Hence, the measured results would increase by about 0.1 in turn, representing a 5% increase in the soil moisture each time (Fig. 3).

**Fig. 4:**
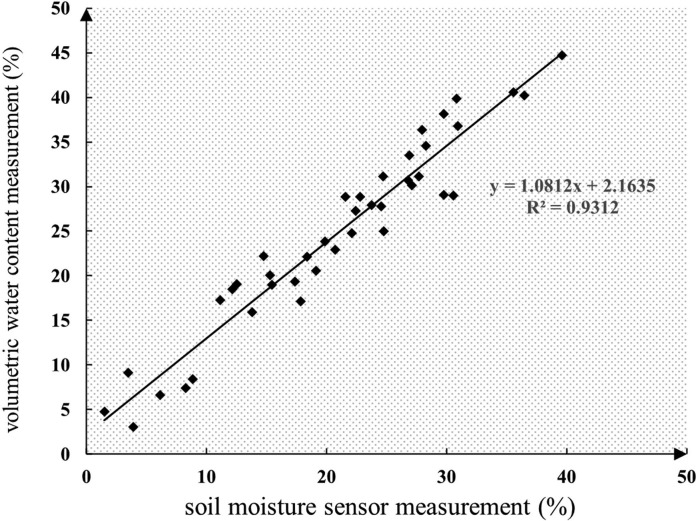
Regression relationship between volumetric water content and volumetric water content measured by the probe.

Through the actual measurement and calibration of the moisture probe, the regression relationship was obtained between the volume moisture content, which is displayed by the probe, and the volume moisture content obtained by the experiment. R2 can reach the value of 0.9312, with a significance of *P*<0.001, as can be observed from Fig. 4.

### Experimental acquisition of soil temperature

The accuracy of the soil temperature sensor was tested by conducting six measurements between −20 °C and 40 °C. From the extracted outcomes, it was demonstrated that at the standard values of −20.3 °C, −5.02 °C, 10.01 °C, 20.04 °C, 30.00 °C, and 40.03 °C, the sensors exhibited soil temperatures of −20.15 °C, −5.08 °C, 10.05 °C, 19.98 °C, 29.87 °C, and 40.06 °C, respectively. The respective errors were 0.15 °C, −0.06 °C, 0.04 °C, −0.06 °C, −0.13 °C, and 0.03 °C, with an average error of 3%.

### Calculation and verification of the soil moisture

**Fig. 5:**
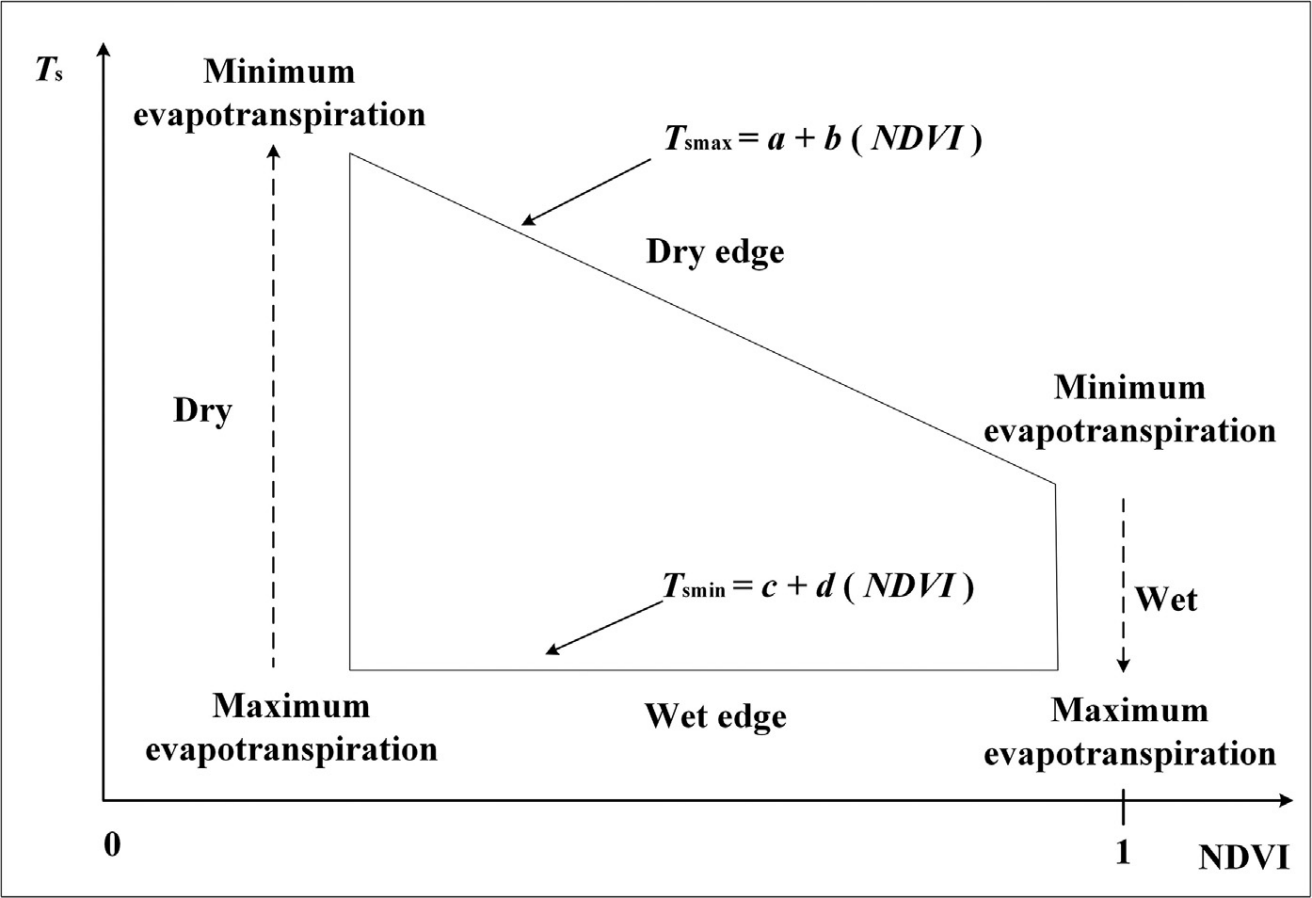
Definition of the TVDI. The TVDI for a given NDVI is estimated by using T_s_, T_smin_, and T_smax_ (see Eq. (1); [5]).

The temperature vegetation dryness index (TVDI) was used and the soil moisture was measured to construct a linear relationship model. The TVDI is a simplified land surface dryness index based on the relationship between the land surface temperature (*Ts*) and the normalized difference vegetation index (*NDVI*). Its basic principle is presented in Fig. 5.

(1) $$\mathrm{TVDI}=\left(T_{s}\;-T_{\mathrm{smin}}\right)/\left(T_{\mathrm{smax}}\;-\;T_{\mathrm{smin}}\right)\;$$

(2) $$T_{\mathrm{smax}}=a\;+\;b\left(NDVI\right)\;$$

(3) $$T_{\mathrm{smin}}=c\;+\;d\left(NDVI\right)\;$$

(4) NDVI=(NIR−R)/(NIR+R)

In Formula (1), Ts refers to the land surface temperature, *T*_*smax*_ forms the dry edge and denotes the maximum surface temperature, and *T*_*smin*_ forms the wet edge and represents the minimum surface temperature. Both *a* and *b* represent the linear fitting coefficient of the dry edge, whereas both c and d refer to the linear fitting coefficient of the wet edge. In Formula (4), *NIR* and *R* refer to the reflectance coefficients of the near-infrared and red bands, respectively. For the calculation of the land surface temperatures from Landsat-5 images, the thermal analysis GEE script developed by Jimenez-Munoz et al. [6] of the Center for Food Safety and Environment, Stanford University was adopted. The Landsat 8 LST and Landsat 9 LST was retrieved by using an algorithm developed by Avdan et al. [7] based on the calculations from the thermal infrared sensor Band 10.

(5) $$LST_{landsat5}=\gamma \left[\varepsilon^{-1}\left(\psi_{1}L+\psi_{2}\right)+\psi_{3}\right]+\delta$$

(6) $$\gamma =T_{b}^{2}/b_{\gamma }L$$

(7) $$\delta =T_{b}-T_{b}^{2}/b_{\gamma }$$

(8) $$\psi_{1}=0.14714\omega^{2}-0.15583\omega +1.1234$$

(9) $$\psi_{2}=-1.1836\omega^{2}-0.37607\omega -0.52894$$

(10) $$\psi_{3}=-0.04554\omega^{2}+1.8719\omega -0.39071$$

(11) $$\mathrm{LS}T_{\mathrm{land}\mathrm{sat}8}=\left(\mathrm{band}10/\left(1+\left(0.00115\left(\mathrm{band}10/1.438\right)\right)\log\left(\mathrm{EM}\right)\right)\right)-273.15$$

(12) $$\mathrm{LS}T_{\mathrm{land}\mathrm{sat}9}=\left(\mathrm{Tb}/\left(1+\left(0.00115\left(\mathrm{Tb}/1.438\right)\right)\log\left(\mathrm{EM}\right)\right)\right)-273.15$$

(13) EM=0.0004FVC+0.986

(14) Tb=1329.2405/log(799.0284/(0.00038Tb+0.1)+1)

(15) $$FVC=\left(NDVI-NDVI_{min}\right)/\left(NDVI_{max}-NDVI_{min}\right)$$

In Formula (5)-(15), *L* is the radiation value, which is carried out according to the observed value of TIR band, Radiation calibration can obtain. *ε* is the emissivity of TIR band, *Tb* is the brightness temperature of TIR band, *ψ1, ψ2* and *ψ3* is the functional parameter of atmosphere and a function of atmospheric water vapor content *ω. EM* is specific irradiance of TIR band. *FVC* is Vegetation coverage.

**Fig. 6:**
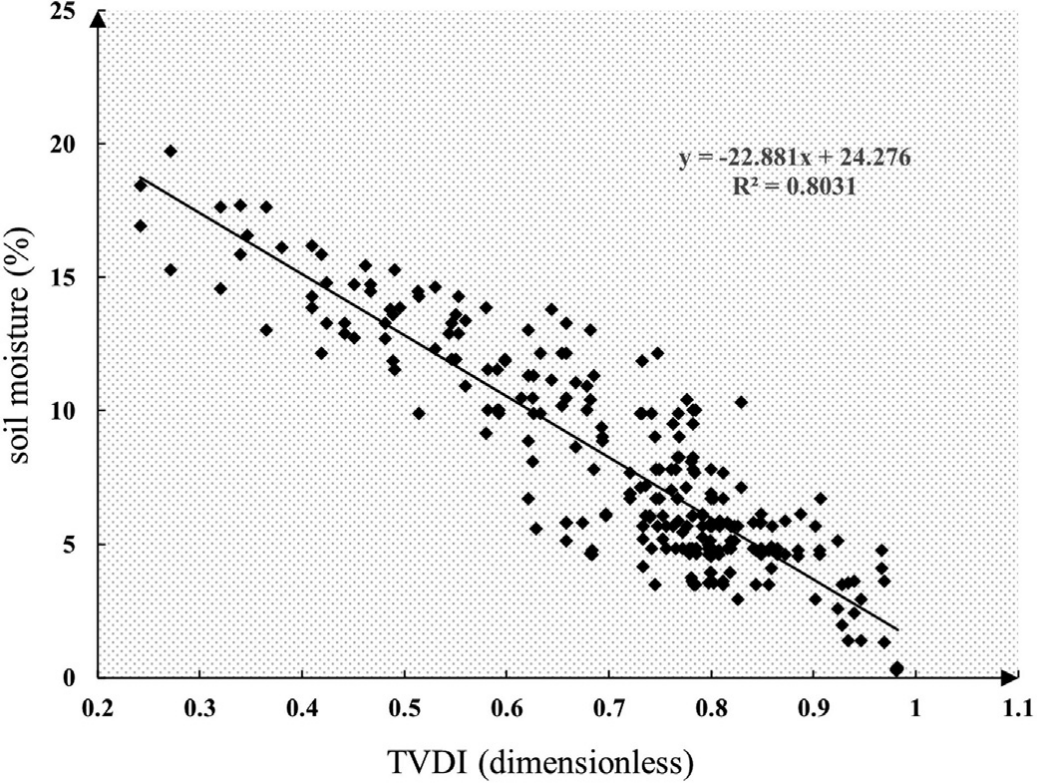
TVDI vs. the measured soil moisture with the linear model fitted.

Fig. 6 depicts the linear relationship between TVDI and the measured soil moisture, which had a root mean square error of 1.89% (*P* < 0.001).

### Calculation method of the land surface temperature and NDVI

To investigate the interannual variation in the soil moisture before and after the wind farms were built, as well as to minimize the influence of the model error and climate change on the research results, the method of Zhou et al. [8] was utilized. More specifically, the difference between the mean soil moisture in each season before (1984–2008) was determined and after the wind farms were built (2009–2022).

### Methods for evaluating spatiotemporal differences in the measured soil moisture

First, the operation of the wind farm was judged based on the wind speed data, to exclude the influence of the difference in surface conditions on the results. Second, the wind position of each automatic weather station was judged based on the wind direction data. As a result, the upwind direction, downwind direction and crosswind direction were obtained. Since the crosswind direction was not on the parallel axis of the wind direction, it was the least affected. Hence, the crosswind direction was taken as the control.

### Wind turbine operational state and division of seasons

Through observation, it was established that the wind turbines started to operate when the wind speeds recorded by the automatic meteorological stations were between 1.4 m/s and 1.8 m/s. Therefore, each 5 min logger record was categorized as either ‘operational’, if the wind speed recorded at all five meteorological monitoring stations was equal, or above 1.8 m/s and ‘stopped’, if all wind speeds were less than 1.4 m/s. In order to thoroughly investigate the seasonal influence of wind turbines on both temperature and humidity, 1 March 2018–31 May 2018 was defined as spring, 1 June 2018–31 August 2018 as summer, 1 September 2018–30 November 2018 as autumn, and 1 December 2018–25 February 2019 as winter [9].

### Interpolation of the dominant wind direction

A wind rose was generated for the operation days, different parts of the day, the full year, and each season from all the data (Appendix Fig. S1). Given the large area of the Huitengliang Mountain wind power base, an interpolation method was used to obtain the dominant wind directions at all locations. Every time, the wind direction values of four meteorological stations were used for spatial interpolation to get the interpolated value (IV) of the remaining meteorological station's wind direction. Then, the relative error (RE) between the interpolated value (IV) and the measured value (MV) was calculated by using formula (16). After comparing the relative errors by using Kriging, the inverse distance weighted (IDW), regulated spline (RS), tension spline (TS), and trend surface (TDS), it was found that the RE values were smallest with the IDW method, with the average error ∼16% (Appendix Table 1). Thereby, the IDW method was used to determine the dominant wind direction on “operation” days and in different periods of the operation days, as can be observed from Fig. S2. Interpolated dominant wind direction charts for the full year and each season were also obtained, as is shown in Fig. S3.

(16) $$RE=\frac{\left|MV-IV\right|}{MV}$$

From the acquired interpolation results, it can be seen that in all seasons and periods, the meteorological station No. 1 was upwind (UW), No. 4 was downwind (DW), No. 5 was the side wind (SW), No. 3 was inside the wind farm (IW), and No. 2 had both side and downwind airflow. Hence, it was named UC (uncertain). In this work, UC wind direction data were not analyzed.

### Minimization of interference factors

To study the influence of wind turbines on soil moisture in all seasons, the wind direction that was unaffected by them needed to be first determined. Although in some works in the literature, upwind measurements as a control point are used for this purpose [[10], [11], [12]], the upwind airflow is also affected by the rotation of the wind turbines [13]. Given the spread of the automatic meteorological stations and the west wind direction in wind roses, it was feasible to use the SW station, which is to the north of the wind power base, as a control. The characteristics of the land surface and any topography could also influence the soil moisture, potentially under or over-estimating any wind farm-derived impacts [14,15]. Therefore, the variability in the soil moisture in each wind direction was calculated when the wind turbines were operational relative to the case when the wind turbines were stopped.

Our meteorological station has the ability to record a piece of data every 5 min. Moreover, the atmospheric temperature and humidity recorded by these meteorological stations were sorted out when the wind speed of all meteorological stations was greater than the value of 1.8 m/s at the same time (operational time). The atmospheric temperature and humidity recorded by each meteorological station were also sorted out when the wind speed of all meteorological stations is less than the value of 1.4 m/s at the same time (stopped time).

To find the differences in the influence of running wind turbines on the soil moisture in different seasons, the following method was applied: in each season, the mean changes of the soil moisture in the UW direction, DW direction, and IW relative to the SW direction at the “stopped time” were subtracted from the mean changes of the soil moisture in the UW direction, DW direction, and IW relative to the SW direction at the “operational time”. The existence of a positive value indicates that the wind turbines induce the soil moisture in this wind direction to increase, and vice versa.

To find the differences in the influence of running wind turbines on the soil moisture in the daytime and at night, after taking into account the variability of the length of the day with the seasons, a day was divided into the following six periods for further analysis: the whole day (0:00 to 24:00), sunrise (04:00 to 08:00), morning (08:00 to 12:00), afternoon (12:00 to 17:00), sunset (17:00 to 20:00), and night (20:00 to 4:00). The employed method can be described as follows: in each period, the mean changes of the soil moisture in the UW direction, DW direction, and IW relative to the SW direction with no “operational time” were subtracted from the mean changes of the soil moisture in the UW direction, DW direction, and IW relative to the SW direction with “operational time”. The manifestation of positive values indicates that the soil moisture in that wind was were increased by the wind turbines, and vice versa. The whole days with “operational time” were considered the “operation” and days, and the whole days with no “operational time” were considered the “stop” days.

## Limitations and uncertainties of the method

The research method of this paper provides a reference for analysing the influence of wind farms on soil moisture. The conclusions provide a reference for further judging the influence of wind farms on local ecosystems. Although the influence extent of a wind farm on soil moisture may be different in different ecosystems and soil types, an overall reduction in soil moisture by wind farms does indeed occur.

## Conclusions

**Fig 7:**
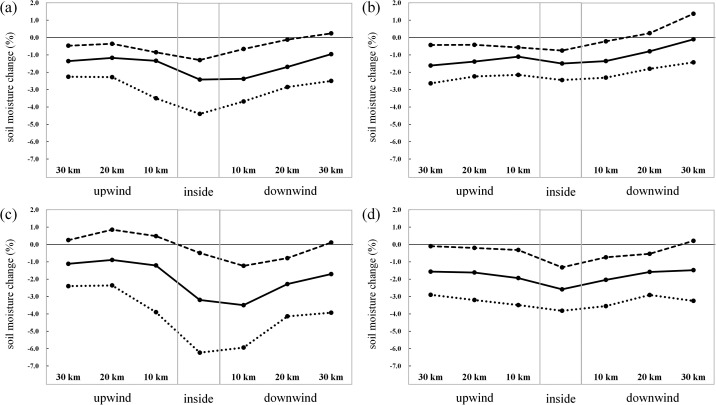
Soil moisture changes in the different areas of the wind farms (the solid line represents the average change value of the soil moisture, the dotted line denotes the minimum change value of the soil moisture, and the dot refers to the maximum change value of the soil moisture) for the full year (a) and in spring (b), summer (c), and autumn (d).

We used remote sensing and field-measured data to analyze changes in soil moisture before and after the construction of wind farms. We found that wind farms significantly reduce soil moisture to different extents according to season and wind direction. We obtained the following main conclusions, as can be observed from Fig. 7:

(1) Wind farms significantly reduced soil moisture within the wind farms and in the upwind and downwind directions. Compared with the upwind and downwind directions, the decrease in soil moisture within the wind farms was the most, and the annual decrease in soil moisture within wind farms reached 4.4%.
(2) Wind farms have different influences on the soil moisture in the upwind and downwind directions in each season. Reductions are greatest in the upwind direction in spring and the downwind direction in summer and autumn.
(3) The wind farm reduced the soil moisture most significantly downwind of the wind farm throughout the day, with an average value of up to 2.85%. The decrease in the soil moisture upwind was the least significant, only by 0.21%.

## Data Availability

Data will be made available on request.

## Appendix Supplementary materials

Image, application 1

Image, application 2
