# Supplementary material for: Wind farms dry surface soil in temporal and spatial variation
Source: MethodsX. 2023 Jan 2;10:102000. doi: 10.1016/j.mex.2023.102000 (PMC9842675; doi:10.1016/j.mex.2023.102000)
Supplement: Supplementary file 1 [file mmc1.docx]

## 1. Measured and meteorological station data

The five automatic meteorological stations are shown in Fig. 1 (NHSF50WS-R, Wuhan Nenghui Technology Co., Ltd.). The depths of the soil temperature and moisture probes were 52 mm (Fig. 2). The soil moisture measurement range was 0–100% (by volume) with an average error of 1% in the range 0–53% and 2% in the range of 53–100%. The temperature measurement range (± error) was −40–80 °C (± 0.4 °C).


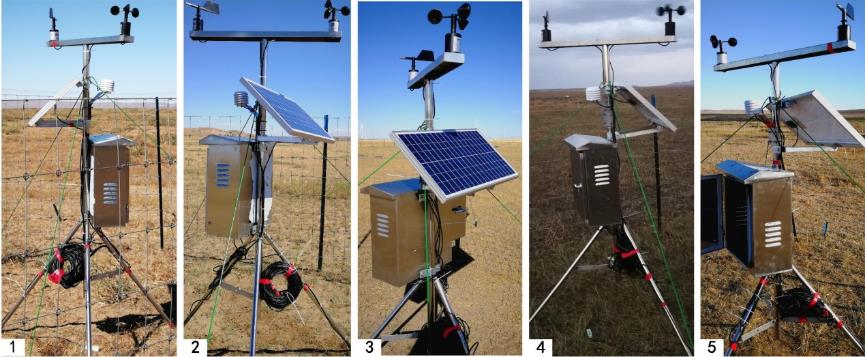


**Fig. 1.** Field photos of the automatic meteorological stations.







**Fig. 2**. Soil temperature and moisture sensor.

## 2. Landsat 5 LST calculation method:

Code: https://code.earthengine.google.com/?scriptPath=users%2Fwanggang5991438%2FLST%3ALD5%20(HTL)

function coreEmissivity(reflimg, NDVImin, NDVImax, Esoil, Eveg){

var ndvi_min = ee.Image(ee.Number(NDVImin));

var ndvi_max = ee.Image(ee.Number(NDVImax));

var ndvi = reflimg.normalizedDifference(["B4", "B3"]);

var fvc = ndvi.subtract(ndvi_min)

.divide(ndvi_max.subtract(ndvi_min))

.pow(ee.Image(2));

var e = ee.Image(0.97).multiply(ee.Image(1).subtract(fvc)).add(ee.Image(0.99).multiply(fvc));

return e.select([0], ['emissivity']);

}

//Convenience function for mapping emissivity computation over collection

function getEmissivity(reflimg){

return coreEmissivity(reflimg, 0.18, 0.85, 0.97, 0.99);

}

//Compute psi functions

function getPsis(joinedimg){

// WTR in NCEP data is in kg/m^2,

// LST method needs g/cm^2: 1 kg/m2 = 10^-1 g/cm^2

// var wv = joinedimg.select('SRWVAP18') //Using individual time-image

// .multiply(ee.Image(0.1)); //conversion to g/cm2

var wv = joinedimg.select('WV_MEAN') // Using average WV

.multiply(ee.Image(0.1)); //conversion to g/cm2

var psi1 = ee.Image(0.14714).multiply(wv.pow(ee.Image(2)))

.add(ee.Image(-0.15583).multiply(wv))

.add(ee.Image(1.1234));

var psi2 = ee.Image(-1.1836).multiply(wv.pow(ee.Image(2)))

.add(ee.Image(-0.37607).multiply(wv))

.add(ee.Image(-0.52894));

var psi3 = ee.Image(-0.04554).multiply(wv.pow(ee.Image(2)))

.add(ee.Image(1.8719).multiply(wv))

.add(ee.Image(-0.39071));

return ee.Image.cat([wv, psi1, psi2, psi3])

.select([0,1,2,3], ["wv_gcm-2", "psi1", "psi2", "psi3"])

.set({'system:time_start':joinedimg.get('system:time_start')});

}

//Compute surface temperature (output in degrees Kelvin)

function getSurfaceTemp(joinedimg){

var brightemp = joinedimg.select('B6_BRT');

var radtemp = joinedimg.select('B6_RAD');

var c1 = ee.Image(1.19104*1e8); // W um^4 m^-2

var c2 = ee.Image(14387.7); // um K

var lambda = ee.Image(11.457); //um (effective wavelength of TM B6)

var beta = ee.Image(1256); //K

var gamma = radtemp.multiply(c2).divide(brightemp.pow(2))

.multiply(radtemp.multiply(lambda.pow(4)).divide(c1)

.add(lambda.pow(-1)))

.pow(-1);

var delta = brightemp.subtract(radtemp.multiply(gamma));

var psis = getPsis(joinedimg);

var e = getEmissivity(joinedimg);

var toctemp = gamma.multiply(psis.select('psi1').multiply(radtemp)

.add(psis.select('psi2'))

.divide(e)

.add(psis.select('psi3')))

.add(delta).subtract(273.15);

var sigma = 5.67e-8; //W/m2/K4

var tocrad = ee.Image(sigma).multiply(e)

.multiply(toctemp.pow(ee.Image(4)))

.divide(ee.Image(Math.PI));

return ee.Image.cat(toctemp, brightemp, e, tocrad, radtemp)

.select([0,1,2,3,4], ['TOCtemp', 'TOAtemp', 'emiss', 'TOCrad', 'TOArad'])

.set({

'DATE_ACQUIRED':joinedimg.get('DATE_ACQUIRED'),

'LANDSAT_SCENE_ID':joinedimg.get('LANDSAT_SCENE_ID'),

'SUN_AZIMUTH':joinedimg.get("SUN_AZIMUTH"),

'SUN_ELEVATION':joinedimg.get("SUN_ELEVATION"),

'system:time_start':joinedimg.get('system:time_start'),

});

}

## 3. Landsat 8 LST calculation method:

Code: https://code.earthengine.google.com/?scriptPath=users%2Fwanggang5991438%2FA001%3Alandsat8_LST

var ndvi = image.normalizedDifference(['B5',

'B4']).rename('NDVI');

var ndviParams = {min: -1, max: 1, palette: ['blue', 'white',

'green']};

print(ndvi,'ndvi');

//Map.addLayer(ndvi.clip(area), ndviParams, 'ndvi');

}

//select thermal band 10(with brightness tempereature), no calculation

var thermal= image.select('B10').multiply(0.1);

var b10Params = {min: 291.918, max: 302.382, palette: ['blue',

'white', 'green']};

//Map.addLayer(thermal.clip(area), b10Params, 'thermal');

// find the min and max of NDVI

{

var min = ee.Number(ndvi.reduceRegion({

reducer: ee.Reducer.min(),

geometry: area,

scale: 30,

maxPixels: 1e9

}).values().get(0));

print(min, 'min');

var max = ee.Number(ndvi.reduceRegion({

reducer: ee.Reducer.max(),

geometry: area,

scale: 30,

maxPixels: 1e9

}).values().get(0));

print(max, 'max')

}

//fractional vegetation

{

var fv =(ndvi.subtract(min).divide(max.subtract(min))).pow(ee.Number(2)).rename('FV');

print(fv, 'fv');

//Map.addLayer(fv);

}

//Emissivity

var a= ee.Number(0.004);

var b= ee.Number(0.986);

var EM=fv.multiply(a).add(b).rename('EMM');

var imageVisParam3 = {min: 0.9865619146722164, max:0.989699971371314};

//LST in Celsius Degree bring -273.15

var LST = thermal.expression(

'(Tb/(1 + (0.00115* (Tb / 1.438))*log(Ep)))-273.15', {

'Tb': thermal.select('B10'),

'Ep': EM.select('EMM')

}).rename('LST');

## 4. Landsat 9 LST calculation method:

Code：https://code.earthengine.google.com/?scriptPath=users%2Fwanggang5991438%2FA001%3Alandsat9%20LST

var col = ld93

.filterBounds(area)

.filter(ee.Filter.eq('WRS_PATH',124))

.filter(ee.Filter.eq('WRS_ROW',30))

.map(maskL8sr)

.filterDate(start,end)

.map(calNDVI)

var thermal= col.select('B10').reduce(ee.Reducer.max());

var b10Params = {min: 2878000, max: 3046000, palette: ['blue', 'white', 'green']};

var ndvi =col.select('NDVI').reduce(ee.Reducer.mean()

// find the min and max of NDVI

{

var min = ee.Number(ndvi.reduceRegion({

reducer: ee.Reducer.min(),

geometry: area,

scale: 30,

maxPixels: 1e9

}).values().get(0));

var max = ee.Number(ndvi.reduceRegion({

reducer: ee.Reducer.max(),

geometry: area,

scale: 30,

maxPixels: 1e9

}).values().get(0));

}

//fractional vegetation

{

var fv = ndvi.subtract(min).divide(max.subtract(min)).rename('FV');

//Map.addLayer(fv);

}

//Emissivity

var a= ee.Number(0.004);

var b= ee.Number(0.986);

var EM=fv.multiply(fv).multiply(a).add(b).rename('EMM');

var imageVisParam2 = {min: 0.98, max: 0.99, palette: ['blue', 'white', 'green']};

var BT = thermal.expression(

'1329.2405/log(799.0284/(bb*0.00038+0.1) + 1)-273.15', {

'bb': thermal,

});

var LST = thermal.expression(

'(Tb/(1 + (0.001145* (Tb / 1.438))*log(Ep)))', {

'Tb': BT,

'Ep': EM.select('EMM')

});

## 5. TVDI calculation method:

code: https://www.cnblogs.com/enviidl/p/16318630.html

pro TVDItask, $

raster1=raster1, $

raster2=raster2, $

TVDI=TVDI, $

minimum=minimum, $

isRaster1Ref=isRaster1Ref, $

step=step, $

resampling=resampling, $

output_Raster=output_Raster,$

display=display

COMPILE_OPT idl2

e =envi(/current)

if raster1.uri eq raster2.uri then begin

void=DIALOG_MESSAGE('The input NDVI and LST raster must be different!',$

/info)

task=envitask('tvditask')

task.raster1=raster1

task.minimum=minimum

task.step=step

task.resampling=resampling

task.display=display

r=e.ui.selecttaskparameters(task)

if r eq 'OK' then task.execute else return

endif

if (TVDI eq 'TVDI') then TVDI=1 else TVDI=0

Channel = e.GetBroadcastChannel()

Abort = ENVIAbortable()

Start = ENVIStartMessage('TVDI-VTCI Task', Abort)

Channel.Broadcast, Start

Progress = ENVIProgressMessage('Executing...' , $

0, Abort)

overLay=GetRasterOverlay( $

raster1, $

raster2, $

isRaster1Ref=isRaster1Ref,$

resampling=resampling)

if n_elements(overLay) ne 2 then return

if isRaster1Ref then refRaster=overLay[0] else refRaster=overLay[1]

rasternew=enviraster( $

uri=output_Raster, $

INHERITS_FROM=refRaster, $

INTERLEAVE='BSQ', $

DATA_TYPE=4)

IF overLay[0].metadata.HasTag('data ignore value') THEN $

dataIgnoreValue=overLay[0].metadata['data ignore value']

tileSize=[2048,2048]

tileSize[0] = overLay[0].ncolumns gt tileSize[0] ? tileSize[0] : overLay[0].ncolumns

tileSize[1] = overLay[0].nRows gt tileSize[1] ? tileSize[1] : overLay[0].nRows

xTiles=overLay[0].CreateTileIterator(tile_Size=tileSize)

yTiles =overLay[1].CreateTileIterator(tile_Size=tileSize)

x=!null

y=!null

for i=0,xTiles.ntiles-1 do begin

percentProgress = Round(i* 100.0/(xTiles.ntile*2))

Progress.Percent = percentProgress

Channel.Broadcast, Progress

IF (Abort.Abort_Requested) THEN BEGIN

Finish = ENVIFinishMessage(Abort)

Channel.Broadcast, Finish

return

BREAK

ENDIF

xTmp=xTiles.next()

yTmp=yTiles.next()

x=[x,xTmp[0:*:step]]

y=[y,yTmp[0:*:step]]

endfor

if isa(dataIgnoreValue,/number) then $

xIndex=where(x ne dataignorevalue and x gt minimum) else $

xIndex=where( x gt minimum)

NanIndex1=finite(x)

NanIndex2=finite(y)

nanIndex=where(NanIndex1 eq 0 or NanIndex2 eq 0)

if nanIndex[0] ne -1 then xindex=setdifference(xIndex,nanIndex)

x=x[xindex]

y=y[xindex]

h=para_tvdi(x,y,minimum=minimum)

_Min=h['Min']

_Max=h['Max']

_mina=_Min[0]

_minb=_Min[1]

_maxa=_Max[0]

_maxb=_Max[1]

xTiles.reset

yTiles.reset

for i=0,xTiles.ntiles-1 do begin

percentProgress = Round((i+xtiles.ntiles)* 100.0/(xTiles.ntile*2))

Progress.Percent = percentProgress

Channel.Broadcast, Progress

IF (Abort.Abort_Requested) THEN BEGIN

Finish = ENVIFinishMessage(Abort)

Channel.Broadcast, Finish

return

BREAK

ENDIF

x=xTiles.next()

y=yTiles.next()

r=cal_TVDI_VTCI(_mina,_minb,_maxa,_maxb,x,y,TVDI=TVDI)

if isa(dataIgnoreValue,/NUMBER) then begin

index=where(x eq dataIgnoreValue)

if index[0] ne -1 then r[index]=dataIgnoreValue

endif

rasternew.settile,r,xTiles

endfor

if TVDI then bnames=['TVDI'] else bnames=['VTCI']

IF rasternew.metadata.HasTag('BAND NAMES') then $

rasternew.metadata.UpdateItem,'BAND NAMES',bnames ELSE $

rasternew.metadata.Additem,'BAND NAMES',bnames

rasternew.save

Finish = ENVIFinishMessage(Abort)

Channel.Broadcast, Finish

if display then begin

View = e.GetView()

Layer = View.CreateLayer(rasternew)

View.Zoom, /FULL_EXTENT

endif

xArr=h['xArr']

minYarr=h['minYarr']

maxYarr=h['maxYarr']

plotWetDry,xArr,minYarr,maxYarr

end
