# Supplementary material for: Wind farms dry surface soil in temporal and spatial variation
Source: MethodsX. 2023 Jan 2;10:102000. doi: 10.1016/j.mex.2023.102000 (PMC9842675; doi:10.1016/j.mex.2023.102000)
Supplement: Supplementary file 2 [file mmc2.docx]

# Appendix:


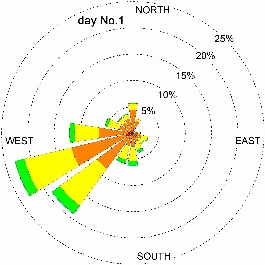

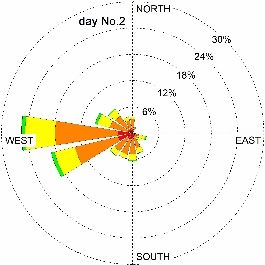

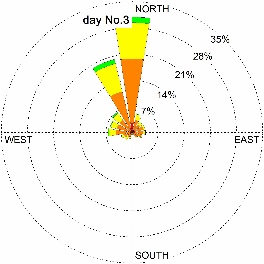

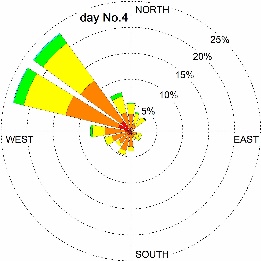

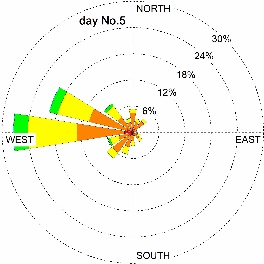


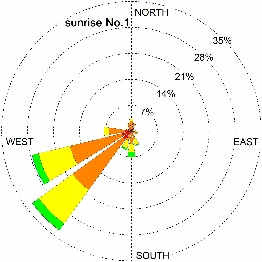

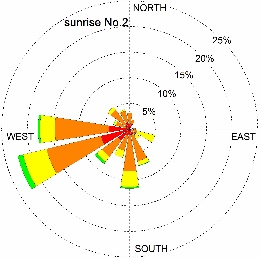

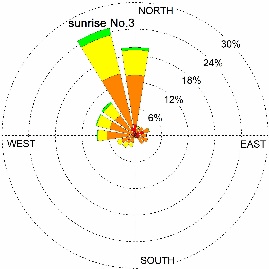

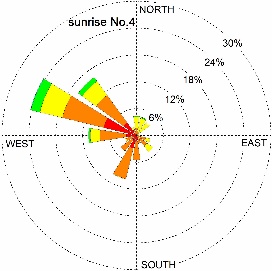

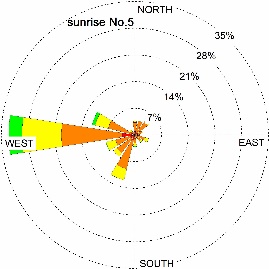


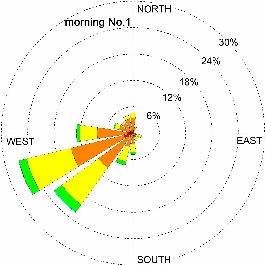

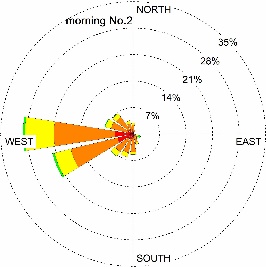

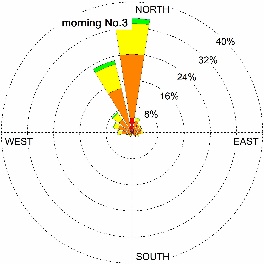

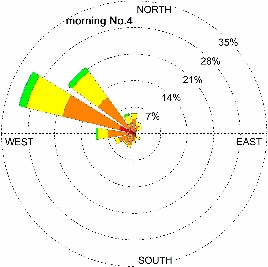

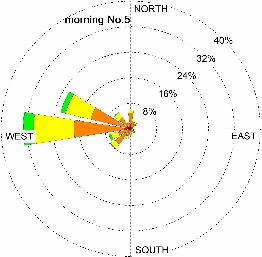


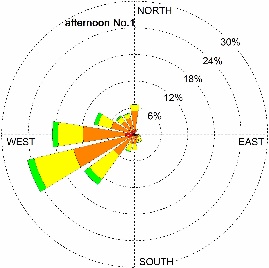

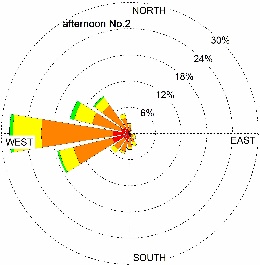

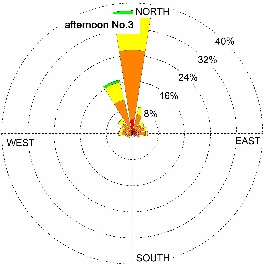

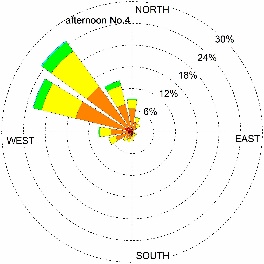

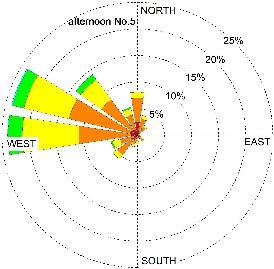


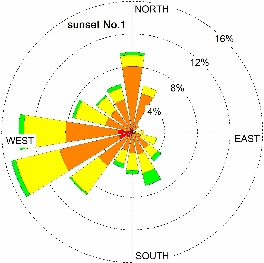

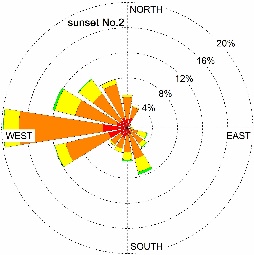

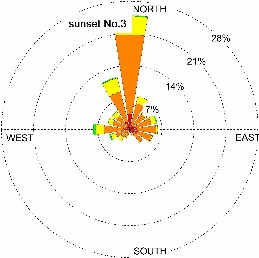

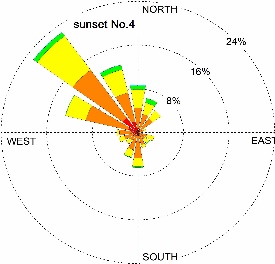

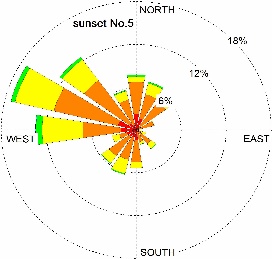


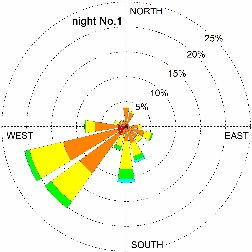

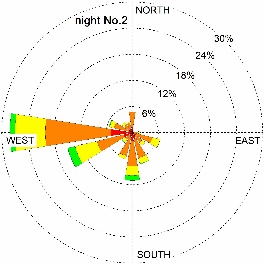

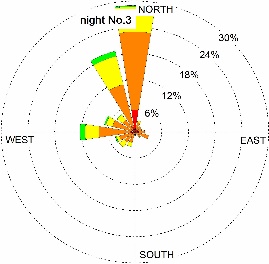

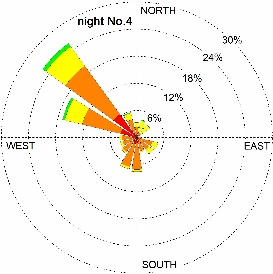

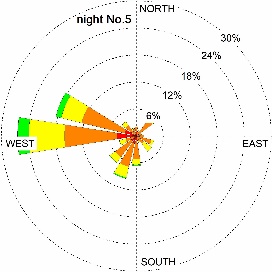


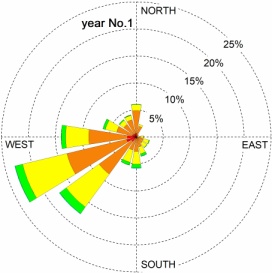

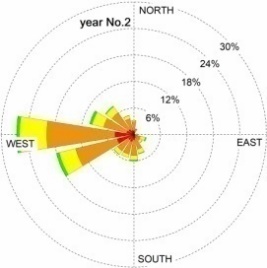

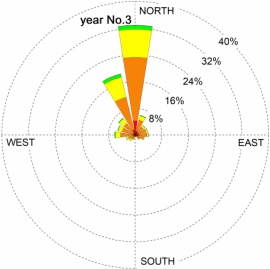

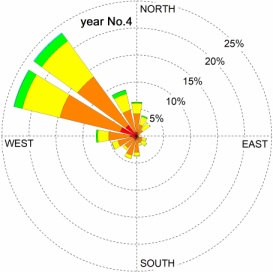

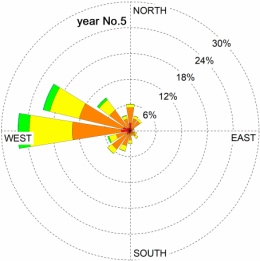


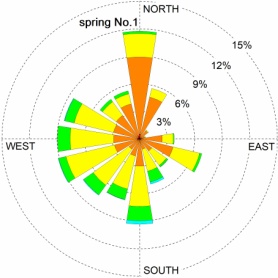

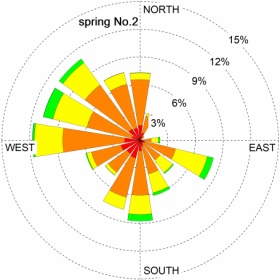

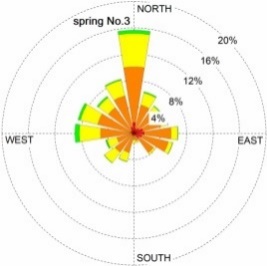

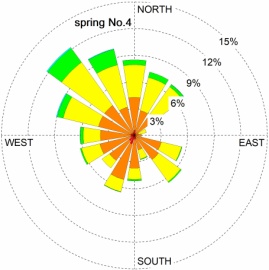

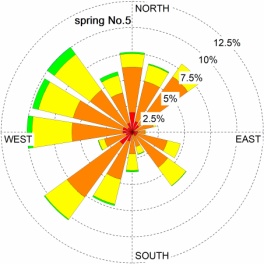


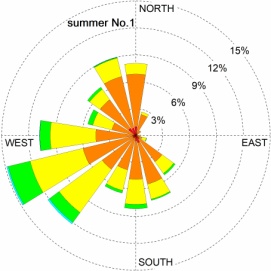

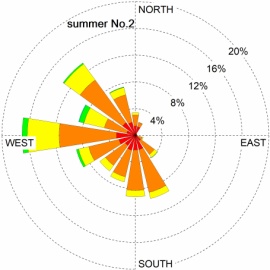

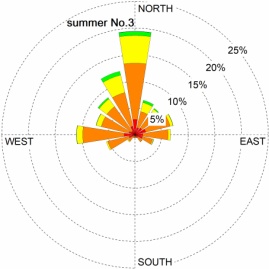

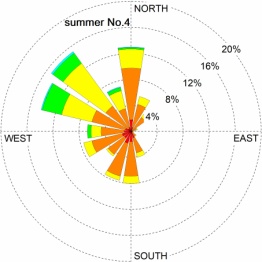

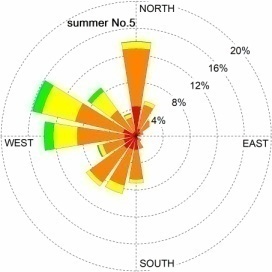


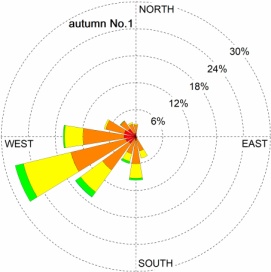

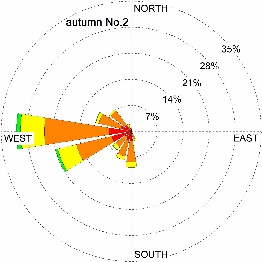

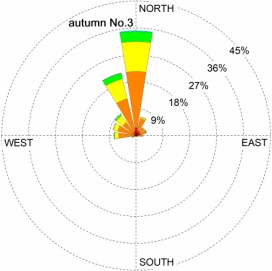

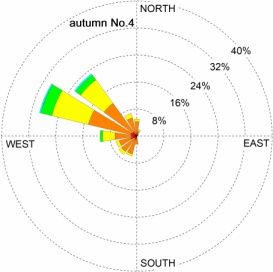

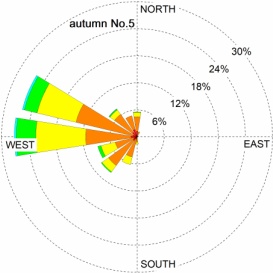


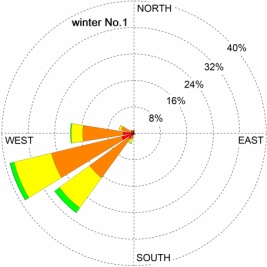

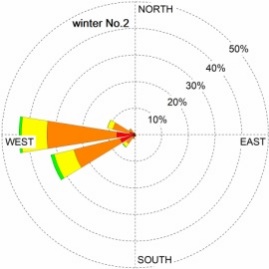

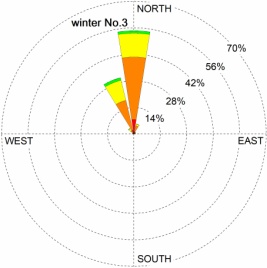

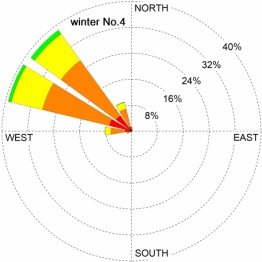

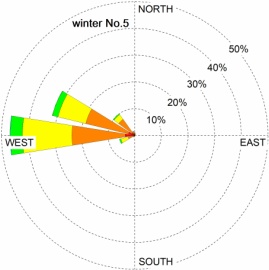

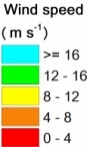


**Fig. S****1.** Wind roses. The first to eleventh rows of all graphs correspond to the operation days, sunrise, morning, afternoon, sunset, night, the full year, spring, summer, autumn, and winter respectively. The first to fifth columns of all graphs denote 1—5 meteorological stations, respectively. The ”day” in the first row of the graphs represents the operation days and the ”year” in the second row of graphs refers to a full year.


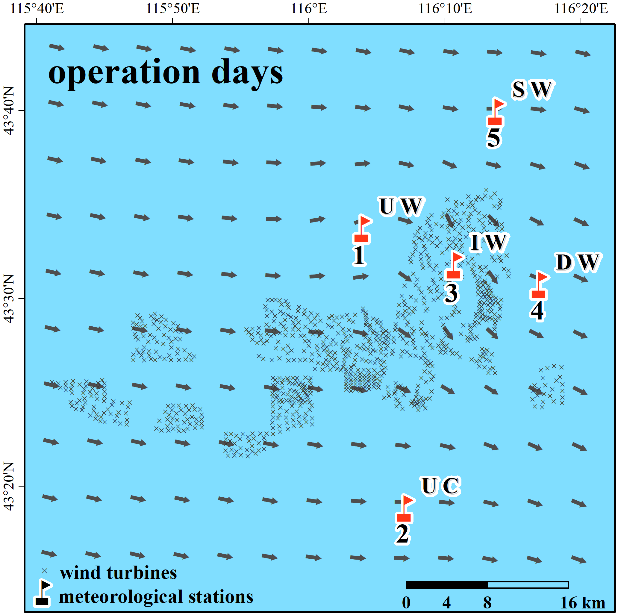

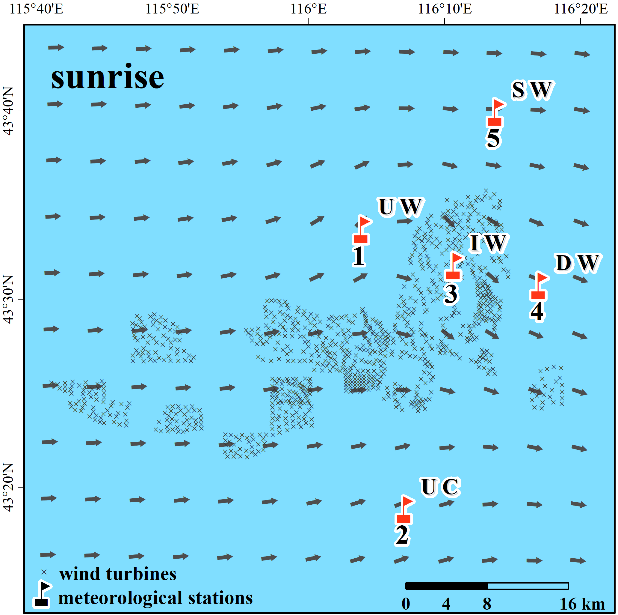

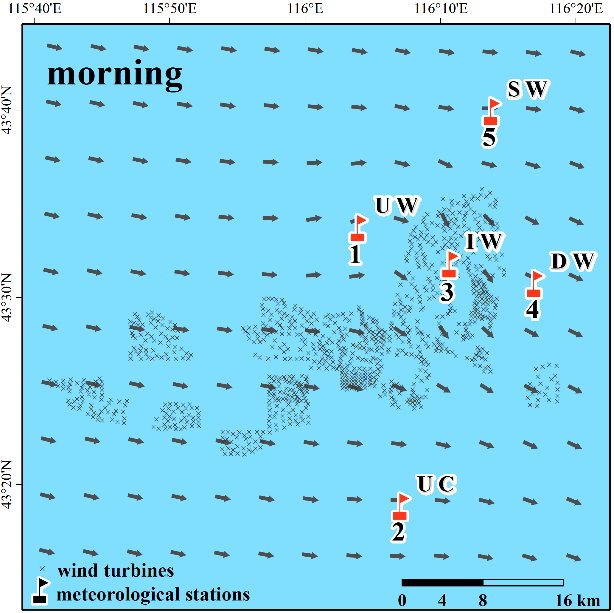


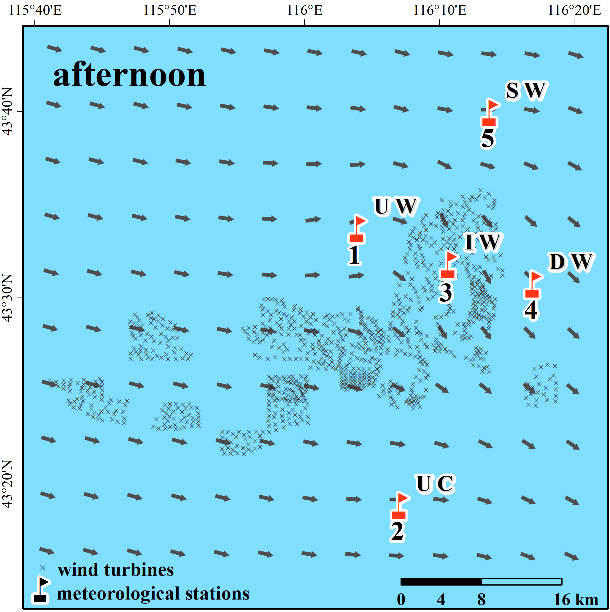

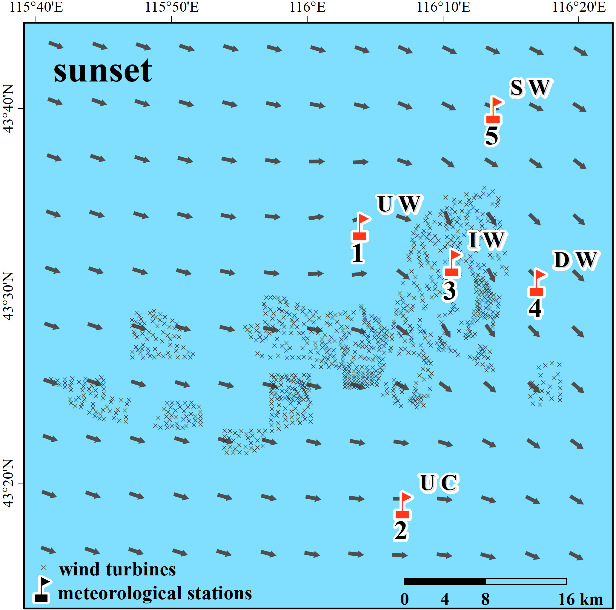

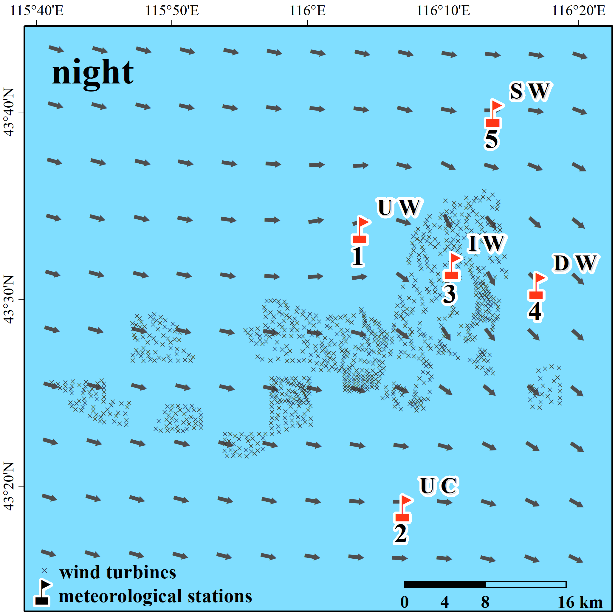


**Fig. S2.** Dominant wind direction on operation days and in different periods of the operation days.


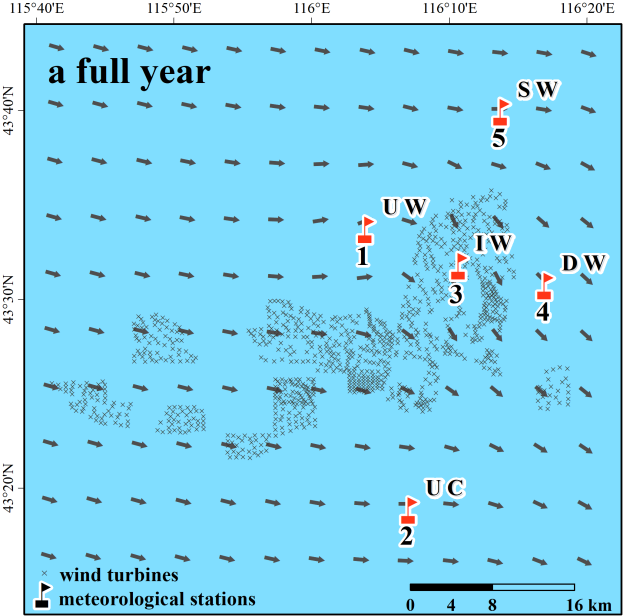

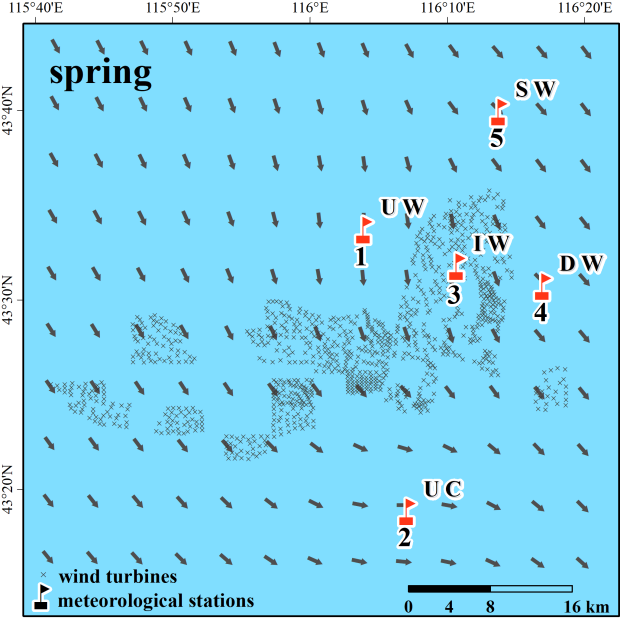

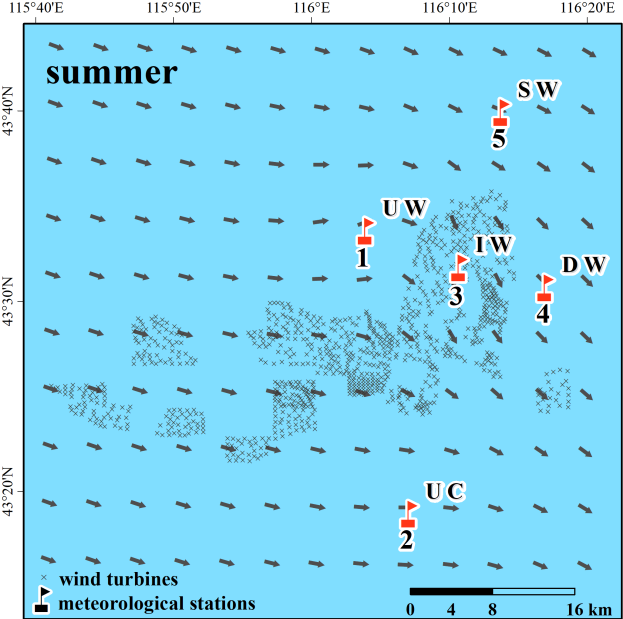

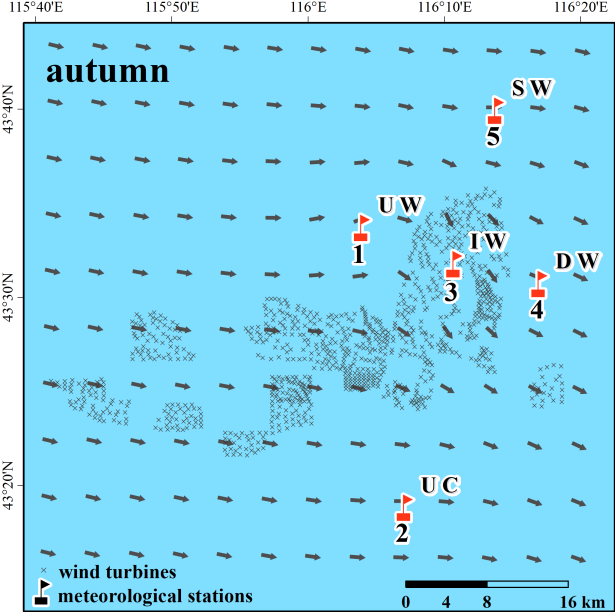

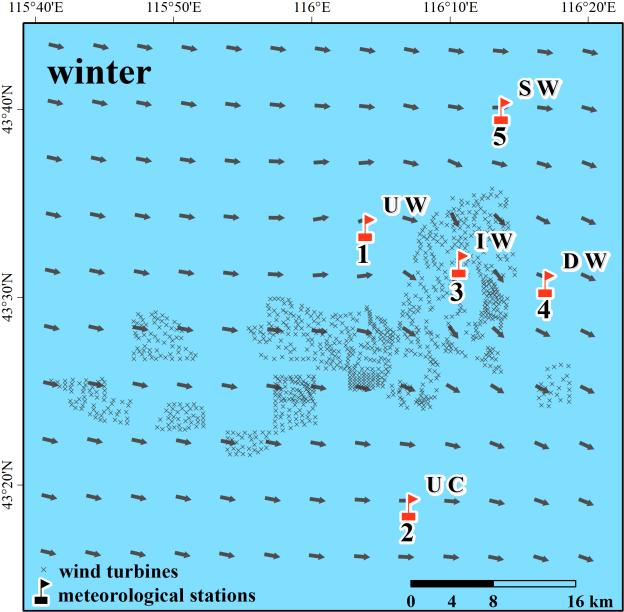


**Fig. S3.** Dominant wind directions in a full year and in all seasons.

**Table S1.** Table of the relative error of the dominant wind direction interpolation method.

| season | Kriging  (%) | IDW（%） | RS  （%） | TS （%） | TDS （%） |
| --- | --- | --- | --- | --- | --- |
| spring | 18 | 12 | 16 | 13 | 19 |
| summer | 23 | 16 | 26 | 22 | 21 |
| autumn | 24 | 18 | 31 | 26 | 25 |
| winter | 24 | 18 | 31 | 26 | 25 |
| a full year | 26 | 17 | 30 | 25 | 23 |
| sunrise | 25 | 17 | 29 | 24 | 23 |
| morning | 24 | 18 | 31 | 26 | 25 |
| afternoon | 26 | 17 | 30 | 25 | 23 |
| sunset | 27 | 16 | 26 | 22 | 21 |
| night | 28 | 17 | 30 | 25 | 23 |
| operation days | 25 | 17 | 30 | 25 | 23 |
| average | 25 | 16 | 28 | 24 | 23 |

IDW = inverse distance weighted; RS= regularized spline;TS = tension spline; TDS= trend surface
